# Individualisierung der antihypertensiven Therapie bei Patient:innen mit Diabetes mellitus. Leitlinie der Österreichischen Diabetes Gesellschaft (Update 2023)

**DOI:** 10.1007/s00508-023-02189-1

**Published:** 2023-04-20

**Authors:** Christoph H. Saely, Gerit-Holger Schernthaner, Johanna Brix, Renate Klauser-Braun, Emanuel Zitt, Heinz Drexel, Guntram Schernthaner

**Affiliations:** 1 VIVIT Institut Feldkirch, Feldkirch, Österreich; 2 Private Universität im Fürstentum Liechtenstein, Liechtenstein, Liechtenstein; 3 Abteilung für Innere Medizin I, Akademisches Lehrkrankenhaus Feldkirch, Feldkirch, Österreich; 4 Klinische Abteilung für Angiologie, Universitätsklinik für Innere Medizin II, Medizinische Universität Wien, Währinger Gürtel 18–20, Wien, Österreich; 5 1. Med. Abteilung mit Diabetologie, Endokrinologie und Nephrologie, Klinik Landstraße, Wien, Österreich; 6 Innere Medizin III, LKH Feldkirch, Feldkirch, Österreich; 7 Landeskrankenhaus Bregenz, Bregenz, Österreich; 8 Drexel University College of Medicine, Philadelphia, PA USA; 9 ESC-Working Group „Cardiovascular Pharmacotherapy“, Sophia Antipolis, Frankreich; 10 Universität Wien, Wien, Österreich; 11 3. Medizinische Abteilung, Klinik Donaustadt, Wien, Österreich

**Keywords:** Hypertonie, Diabetes, Blutdruckzielwerte, Individualisierung, Vaskuläre Komplikationen, Hypertensin, Diabetes, Target values, Individualisation, Vascular complications

## Abstract

Hypertonie ist eine sehr häufige Komorbidität bei Patient:innen mit Diabetes mellitus, die – wenn unzureichend behandelt – signifikant zur erhöhten Mortalität und zum Auftreten von mikrovaskulären und makrovaskulären Komplikationen beiträgt. Eine Individualisierung der Blutdruckzielwerte in Abhängigkeit vom Patient:innenalter und vom Vorliegen bestimmter vaskulärer Komplikationen wird heute weltweit diskutiert. Blutdruckzielwerte um 130/80 mm Hg waren in den Studien mit der geringsten Ereignisrate an Komplikationen assoziiert, wobei die Blutdruckzielwerte je nach Alter und Komorbiditäten individualisiert werden sollten; am wichtigsten ist für die meisten Patient:innen, dass ein Blutdruck < 140/90 mm Hg erreicht wird. ACE-Hemmer oder Angiotensin-Rezeptorblocker sollen in der Hypertonie-Therapie bei Patient:innen mit Diabetes mellitus bevorzugt werden, vor allem wenn Albuminurie oder KHK vorliegen. Für die meisten Patient:innen mit Diabetes ist eine Kombinationstherapie notwendig, wobei Medikamente mit nachgewiesenem kardiovaskulärem Nutzen (neben ACE-Hemmern und altenativ Angiotensin-Rezeptorblockern, Dihydropyridin-Calciumantagonisten und Thiazid-Diuretika) eingesetzt werden sollten, präferentiell als Kombinationspräparate. Nach Erreichung der Zielwerte muss die antihypertensive Therapie fortgeführt werden, wobei regelmäßige Blutdruckmessungen durch die Patient:innen für die Optimierung der Blutdruckeinstellung sehr hilfreich sind. Neuere Antidiabetika wie SGLT2-Inhibitoren oder GLP1-Rezeptoragonisten tragen ebenfalls zur Blutdrucksenkung bei.

## Abstract (en)

Hypertension is one of the most important comorbidities of diabetes, contributing significantly to death and leading to macrovascular and microvascular complications. When assessing the medical priorities for patients with diabetes, treating hypertension should be a primary consideration. In the present review practical approaches to hypertension in diabetes, including individualized targets for preventing specific complications are discussed according to current evidence and guidelines. Blood pressure values of about 130/80 mm Hg are associated with the best outcome; most importantly, at least blood pressure values < 140/90 mm Hg should be achieved in most patients. Angiotensin converting enzyme inhibitors or angiotensin receptor blockers should be preferred in patients with diabetes, especially in those who also have albuminuria or coronary artery disease. Most patients with diabetes require combination therapy to achieve blood pressure goals; agents with proven cardiovascular benefit should be used (including, besides angiotensin converting enzyme inhibitors and alternatively angiotensin receptor blockers, dihydropyridin-calcium antagonists and thiazide diuretics), preferable in single-pill combinations. Once the target is achieved, antihypertensive drugs should be continued. Newer antidiabetic medications such as SGLT-2-inhibitors or GLP1-receptor agonists have also antihypertensive effects.

## Definition

Eine arterielle Hypertonie soll dann diagnostiziert werden, wenn der Blutdruck bei wiederholten Messungen an verschiedenen Tagen ≥ 140/90 mm Hg liegt. Es wurden in den USA niedrigere Grenzwerte für die Diagnose einer Hypertonie vorgeschlagen [1]; aufgrund der fraglichen therapeutischen Konsequenz wird diese Definition allerdings kontrovers diskutiert [2–4]. Die vorliegenden österreichischen Leitlinien [5–7] definieren eine arterielle Hypertonie bei Diabetes mellitus übereinstimmend mit mehreren internationalen Leitlinien als einen Blutdruck ≥ 140/90 mm Hg.

## Epidemiologie und screening

Die arterielle Hypertonie ist eine sehr häufige Komorbidität sowohl bei Patient:innen mit Typ 2 als auch bei jenen mit Typ 1 Diabetes mellitus, und ein erhöhter Blutdruck ≥ 140 mm Hg systolisch und/oder ≥ 90 mm Hg diastolisch findet sich in Abhängigkeit von Diabetesdauer und Vorliegen bzw. Ausmaß einer Adipositas in 50–60 % aller Patient:innen [8]. Der Blutdruck sollte bei jeder Visite eines Patient:innen mit Diabetes kontrolliert werden. Hypertensive Patient:innen mit Diabetes sollten ihren Blutdruck auch zuhause selbst regelmäßig kontrollieren. Wichtig ist eine korrekte Messtechnik: Sitzend, den Arm auf Herzniveau aufgestützt, den Rücken angelehnt, die Beine am Boden, nach 5 min Ruhe mit einer dem Armumfang angepassten Manschettenbreite. Häufig geht die Hypertonie dem Diabetes zeitlich voraus und findet sich dabei assoziiert mit anderen Komponenten des Insulinresistenzsyndroms; Patienten mit Hypertonie haben ein deutlich erhöhtes Risiko, einen Diabetes zu entwickeln [9]. Sie sollen deshalb auf das Vorliegen einer Glukosestoffwechselstörung gescreent werden.

Im Vergleich zu Patient:innen ohne Diabetes findet man insbesondere bei älteren Diabetes-Patient:innen häufig eine isolierte systolische Hypertonie. Das häufige Fehlen einer physiologischen Nachtabsenkung („Dipping“) geht mit einem erhöhten Risiko für Mikroalbuminurie bzw. Linksventrikelhypertrophie einher und ist ein ungünstigster Prädiktor für kardiovaskuläre Ereignisse [7].

## Lebensstilinterventionen zur Blutdrucksenkung

Lebensstilmaßnahmen sind die Basis jeder antihypertensiven Therapie. Bereits bei Patient:innen mit Blutdruckwerten > 120/80 mm Hg sollten eine Gewichtsreduktion bei Übergewicht oder Adipositas, eine Kochsalz-arme und Kalium-reiche Diät, der Verzicht auf übermäßigen Alkoholkonsum sowie körperliche Aktivität empfohlen werden. In der Initialphase der LOOK-AHEAD-Studie (*n* = 5145) senkte eine Gewichtsreduktion von zirka 8 kg nach einem Jahr bei übergewichtigen/adipösen Patient:innen mit Typ 2 Diabetes die Blutdruckwerte um 5/2 mm Hg signifikant [10]; bei Patient:innen mit einem Gewichtsverlust > 15 % betrug die systolische Blutdrucksenkung sogar 10 mm Hg.

## Individualisierung des Zielblutdruckes bei Patient:innen mit Diabetes mellitus?

In epidemiologischen Studien fand sich ein enger Zusammenhang zwischen dem Auftreten von makrovaskulären Komplikationen wie koronarer Herzkrankheit (KHK), Schlaganfall, peripherer arterieller Verschlusskrankheit (PAVK) sowie mikrovaskulären Komplikationen (diabetische Nierenerkrankung und diabetische Retinopathie) und den erhobenen Blutdruckwerten [8]. Da in epidemiologischen Analysen das niedrigste Risiko bei Blutdruckwerten von 120/80 mm Hg beobachtet wurde [11, 12] glaubte man, dass möglichst niedrige Blutdruckwerte mittels antihypertensiver Therapiemaßnahmen bei Patient:innen zu erzielen wären.

Bis 2007 wurde deshalb in Guidelines ein systolischer Zielblutdruck bei Patient:innen mit Diabetes mellitus unabhängig vom Alter < 130 mm Hg gefordert [13], wobei für Patient:innen mit diabetischer Nierenerkrankung sogar Zielwerte unter 120/75 mm Hg empfohlen wurden, obwohl Evidenz-basierte Interventionsstudien für diese Zielwerte nie vorlagen.

In weiterer Folge publizierte Interventionsstudien bestätigten allerdings die Annahme eines generellen Vorteils sehr niedriger Blutdruckwerte nicht [14–17]. Im Blutdruckarm der ACCORD-Studie wurden Nachteile einer zu niedrigen Blutdruckeinstellung (< 120 mm Hg) nachgewiesen: Es wurden hier 4700 Patient:innen mit Typ 2 Diabetes entweder einer intensivierten Therapie (systolischer Zielblutdruck < 120 mm Hg) oder einem Standardtherapiearm (systolischer Zielblutdruck < 140 mm Hg) zugeordnet [18]. Nach ca. 5 Jahren betrugen die Blutdruckwerte 119 mm Hg versus 133 mm Hg. Durch die intensivierte Therapiestrategie wurde der primäre Endpunkt schwerer kardiovaskulärer Ereignisse nicht signifikant gesenkt, auch die Mortalität wurde nicht beeinflusst; Schlaganfälle nahmen allerdings um 47 % ab. Die intensivierte Therapie war allerdings mit kardialen Arrhythmien, Hyperkaliämie und Hypotension assoziiert. Subsets der Patient:innen in ACCORD mit hohem kardiovaskulärem Risiko profitierten allerdings von einer aggressiven Blutdruckeinstellung [19, 20]. Ähnlich wie bei der Intensität der Glukoseeinstellung [21] scheint es also sinnvoll, die Intensität der antihypertensiven Therapie individuell auf den Patient:innen anzupassen [22].

Dabei muss aber vorsichtig vorgegangen werden. In einer observationellen Studie von Cooper-DeHoff et al. [23] fand sich etwa bei Patient:innen mit Diabetes und koronarer Herzerkrankung unter einer intensivierten Blutdrucksenkung eine Zunahme der Gesamtmortalität; bei Patient:innen mit systolischen Blutdruckwerten < 110 mm Hg war die Mortalität im Vergleich zu Patient:innen mit systolischen Blutdruck-Werten von 125–130 mm Hg signifikant erhöht. Auch in der VADT-Studie fand sich eine signifikante Zunahme der kardiovaskulären Ereignisse, wenn die diastolischen Blutdruck-Werte < 70 mm Hg abgesenkt wurden [24].

## Heterogene Blutdruckzielwerte verschiedener Fachgesellschaften

Die aktuellen Leitlinien der American Diabetes Association (ADA) 2022 [25] fordern ein generelles Blutdruckziel von < 140/90 mm Hg, niedrigere Blutdruckziele < 130/80 mm Hg können bei hohem kardiovaskulärem Risiko erwogen werden, wenn das Erreichen dieser Werte adäquat vertragen wird. Die Guidelines von American College of Cardiology/American Heart Association empfehlen hingegen Blutdruckzielwerte von < 130/80 mm Hg bei Patient:innen mit und bei jenen ohne Diabetes mellitus [1]; Blutdruckzielwerte von < 130/80 mm Hg werden auch von Kanadischen Experten [26], von Japanischen Experten [27] sowie von IDF und WHO [7] empfohlen. UK-NICE empfiehlt einen Zielblutdruck < 140/90 mm Hg bei Patient:innen < 80 und von < 150/90 mm Hg bei Patient:innen ≥ 80 Jahren [28].

Die 2021 publizierten Guidelines der ESC zur kardiovaskulären Prävention [29] empfehlen als erstes Ziel einen Blutdruck von zumindest < 140/90 mm Hg für alle Patient:innen, letztlich aber für Patient:innen < 70 Jahre ein systolisches Blutdruckziel zwischen 120 und 130 und für Patient:innen ≥ 70 Jahre ein systolisches Blutdruckziel < 140 mm Hg und bis zu 130 mm Hg, wenn die Blutdrucksenkung gut vertragen wird; für alle Patient:innen wird ein diastolisches Blutdruckziel < 80 mm Hg postuliert. Es wird hier nicht zwischen Patient:innen mit und Patient:innen ohne Diabetes differenziert. Ältere Guidelines erlaubten auch höhere Zielwerte [30, 31].

## Warum ist das alles so kompliziert?

Die unterschiedlichen Empfehlungen basieren darauf, dass die Studienlage hinsichtlich intensivierter Blutdrucksenkung komplex ist und widersprüchlich interpretiert wird, wobei dies insbesondere für die Studien ACCORD-BP (bei Patient:innen mit Diabetes) [18] und SPRINT (bei Studienteilnehmern ohne Diabetes) [32] gilt. In beiden Studien wurden im intensivierten Studienarm Blutdruckzielwerte < 120/80 mm Hg verfolgt und mit den Ergebnissen der Kontrollkohorten (Zielwert < 140/80 mm Hg) verglichen. Während in der ACCORD-BP-Studie der kardiovaskuläre Tod durch die intensivierte Blutdruckkontrolle nicht gesenkt wurde (HR 1,06; 95 % CI 0,74–1,52), fand sich in der SPRINT (0,57; 0,38–0,85) Studie eine dramatische Senkung. Auch die Risikobeeinflussung für die Herzinsuffizienz war unterschiedlich: ACCORD-BP (0,94; 0,70–1,26), und SPRINT (0,62; 0,45–0,84).

Es ist unklar, ob die unterschiedlichen Studienergebnisse auf die differenten Populationen zurückzuführen sind, oder ob auch das Studiendesign einen Einfluss hatte [33]. Auch die unterschiedliche Blutdruckmessung und die limitierte statistische power (die 95 % Konfidenzintervalle überlappen sich) müssen dabei bedacht werden. Bei der unbeobachteten automatisierten Messung in SPRINT sind insgesamt niedrigere Werte zu erwarten, die wahrscheinlich höheren in der Selbstmessung und bei der Ordinationsmessung entsprechen.

In der ACCORD-Studie wurde gleichzeitig auch der Effekt einer intensivierten Blutzuckerkontrolle (HbA_1c_-Zielwert < 6,0 %) untersucht, wobei in der intensivierten Gruppe eine erhöhte kardiovaskuläre Mortalität beobachtet wurde [32]. In einer Re-Analyse der ACCORD-Studie [34] fand sich im glykämischen Standardarm unter intensivierter Blutdrucksenkung eine signifikante Senkung von Schlaganfällen (−39 %), Myokardinfarkten (−37 %) und CV-Endpunkten insgesamt (−33 %); Mancia und Grassi [4] diskutieren einen potenziell negativen Effekt von schweren Hypoglykämien im intensivierten Blutzuckerarm auf den potenziell vorteilhaften Effekt einer intensivierten Blutdruckeinstellung.

In einer gepoolten Analyse von ACCORD-BP und SPRINT fanden sich ähnlich günstige Effekte auf kardiovaskuläre Endpunkte unter einer intensivierten Blutdruckkontrolle mit fehlender statistischer Heterogenität [20] In einer weiteren gepoolten Sekundäranalyse von ACCORD-BP und SPRINT fanden sich hingegen warnende Hinweise darauf, dass eine zu strikte Blutdruckeinstellung die Inzidenz der chronischen Niereninsuffizienz (CNI) steigern dürfte [35]: Bei Patient:innen ohne CNI vor Intervention (*n* = 4311 in ACCORD; *n* = 6715 in SPRINT), wurde der Effekt der systolischen Blutdrucksenkung (13,9 mm Hg in ACCORD und 15,2 mm Hg in SPRINT) auf einen Abfall der eGFR > 30 % auf < 60 mL/min per 1,73 m^2^ analysiert. Nach drei Jahren betrug die kumulative Inzidenz einer CNI in der ACCORD-Studie 10 % im intensivierten Arm vs. 4,1 % im Standardarm; auch in der SPRINT-Studie war die Inzidenz einer CNI signifikant höher im intensivierten Arm: 3,5 % vs. 1,0 % im Standardarm. Eine Überwachung der Nierenfunktion unter intensivierter Blutdruckkontrolle ist daher sehr wichtig.

Eine große Kohortenstudie [36] spricht für ein Blutdruckziel < 140 mm Hg auch bei Patient:innen mit Typ 2 Diabetes ohne etablierte kardiovaskuläre Erkrankungen; niedrigere systolische Werte < 130 oder < 120 mm Hg waren mit keinem zusätzlichen kardiovaskulären Vorteil assoziiert. Patient:innen mit erreichten Blutdruck-Werten < 120 mm Hg hatten im Gegenteil ein signifikant höheres Risiko als Patient:innen mit Werten < 130 mm Hg (Hazard Ratio (HR) 1,75 [95 % Confidence Intervall [CI]: 1,53–2,00]) oder < 140 mm Hg (HR 1,67 [95 % CI: 1,46–1,90]). Bei jüngeren Patient:innen (< 65 Jahre) war das Risiko allerdings signifikant geringer, wenn Blutdruckwerte < 130 mm Hg erzielt wurden (HR 0,81 [95 % CI: 0,69–0,96]) im Vergleich zu Patient:innen mit Blutdruckwerten < 140 mm Hg. Bei älteren Patienten (> 65 Jahre) hingegen war kein Vorteil für Blutdruckwerte < 130 mm Hg zu beobachten.

Bezüglich der Zielwerte für den diastolischen Blutdruck besteht weniger Diskrepanz der Expertenmeinung. In der HOT (Hypertension Optimal Treatment) Studie fand sich bei Typ 2 Diabetes-Patient:innen [37] mit diastolischen Blutdruckzielwerten < 80 mm Hg (erreichter Zielwert betrug 81 mm Hg) eine 51 % Senkung der CV-Ereignisse im Vergleich zur Kontrollgruppe (< 85 mm Hg oder < 90 mm Hg). In der UKPDS-Studie [38] führte die Senkung des diastolischen Blutdruckes von 87 auf 82 mm Hg zu einer signifikanten Abnahme von Myokardinfarkt (−21 %) und Schlaganfall (−44 %). In der SAVOR-Studie [39] fand sich die niedrigste CV-Ereignisrate bei diastolischen Blutdruckwerten zwischen 80 und 90 mm Hg und systolischen Blutdruckwerten zwischen 130 und 140 mm Hg. Diastolische Blutdruckwerte < 60 mm Hg waren mit einem erhöhten Myokard-Infarkt-Risiko assoziiert (HR 2,30 [95 % CI 1,50–3,53]) im Vergleich zu Patient:innen mit höheren diastolischen Blutdruckwerten (80–90 mm Hg). Die ADA Guidelines [25] empfehlen diastolische Blutdruckwerte < 90 mm HG die ESC Guidelines zur kardiovaskulären Prävention [29] empfehlen diastolische Blutdruckwerte von < 80 mm Hg.

## Therapie mit Antihypertensiva

Bei bestätigter Hypertonie (Blutdruck ≥ 140/90 mm Hg bei mehreren Messungen an verschiedenen Tagen) sollte eine medikamentöse Blutdrucktherapie begonnen und zeitnahe angepasst werden. Liegt der Blutdruck ≥ 160/100 mm Hg, so sollte bereits initial eine Kombination von zwei antihypertensiven Wirkstoffen gegeben werden. Es sollten Antihypertensiva verwendet werden, für die bei Diabetes eine Reduktion von kardiovaskulären Ereignissen gezeigt wurde: ACE-Hemmer, Angiotensin-2-Rezeptorblocker, Diuretika vom Thiazid-Typ und Calciumantagonisten vom Dihydropyridin-Typ. Die aktuellen ESC Leitlinien zur kardiovaskulären Prävention [29] empfehlen einen ACE Hemmer oder Angiotensin Rezeptor Blocker unabhängig vom Vorliegen eines Diabetes als Antihypertensivum der ersten Wahl, die aktuellen Leitlinien der ADA [25] empfehlen einen ACE Hemmer bzw. Angiotensin Rezeptor Blocker bei allen Diabetes-Patient:innen mit Albuminurie oder KHK. Die meisten Patienten mit Diabetes benötigen eine Kombinationstherapie; ACE Hemmer bzw. Angiotensin Rezeptor Blocker wirken potent in der Kombination mit Diuretikum und Kalziumkanalblocker. Sie werden in der vorliegenden Leitlinie als antihypertensive Wirkstoffe der ersten Wahl empfohlen.

Bei Patient:innen mit signifikanter Albuminurie (Albumin/Kreatinin-Ratio ≥ 30 mg/g) sollte ein ACE-Hemmer oder ein Angiotensin-Rezeptorblocker (ARBs) in der höchsten für die Hypertoniebehandlung zugelassenen und verträglichen Dosierung gegeben werden. Dies gilt nicht nur für hypertensive Patienten: Unabhängig vom Blutdruck sollten ARBs oder ACE-Hemmer bei Patient:innen verwendet werden, die eine Albuminurie aufweisen. Die Blutdruckzielwerte müssen allerdings entsprechend dem Alter, dem Pulsdruck, einer vorbestehenden koronaren Herzerkrankung, einer PAVK, Risiko der Progression der Nierenerkrankung sowie Abwesenheit oder Vorliegen einer diabetischen Retinopathie individualisiert werden.

Unter Behandlung mit einem ACE-Hemmer, einem Angiotensin-2-Rezeptorblocker oder einem Diuretikum sollte die Nierenfunktion zumindest einmal im Jahr kontrolliert werden.

Bei Diabetes-Patient:innen mit Nierenerkrankung ist es besonders schwierig die Blutdruckzielwerte zu erreichen, sodass bei der Mehrzahl aller Diabetes-Patient:innen eine antihypertensive Kombinationstherapie erforderlich ist.

Die Kombination von ACE-Hemmern und Angiotensin-Rezeptorblockern ist zur Blutdrucksenkung nicht sinnvoll, eine Kombination dieser beiden Medikamentenklassen mit direkten Renin-Inhibitoren ist kontraindiziert. Es wurde in der Vergangenheit versucht, durch eine Doppelblockade des Renin-Angiotensin-Aldosteron-Systems die Progression der Albuminurie besser zu beeinflussen als mit einer Monotherapie mittels ACE-Hemmer, ARB oder Renin-Inhibitoren. Bei diesen Studien – ONTARGET [40, 41], ALTITUDE [42] – zeigte sich zwar eine stärkere Reduktion der Albuminurie durch die duale Blockade, schwere Nebenwirkungen (unter anderem Hypotonie und Hyperkaliämie) wurden allerdings unter dieser Kombinationstherapie signifikant häufiger beobachtet. Auch bei diesen Studien waren wahrscheinlich RR-Werte deutlich unter 130 mm Hg systolisch für das Auftreten von schwerwiegenden Ereignissen, sowie dem schlechteren Outcome bei Patient:innen mit zusätzlicher KHK zumindest mitverantwortlich.

Lange Zeit wurden Kombinationstherapien von ACE-Inhibitoren oder ARB mit niedrigdosierten Diuretika favorisiert. In der ACCOMPLISH-Studie [43] wurde allerdings bei 6000 Patient:innen mit Typ 2 Diabetes und Hypertonie nachgewiesen, dass bei gleichen Blutdruckzielwerten (135/80 mm Hg) die Kombination von ACE-Inhibitoren mit Amlodipin der Kombination von ACE-Inhibitoren und Hydrochlorothiazid deutlich überlegen war. Die Überlegenheit könnte aber auch darauf zurückzuführen sein, dass die Halbwertszeit von Amlodipin wesentlich länger ist als jene von Hydrochlorothiazid. Da Diabetes-Patient:innen häufig – insbesondere, wenn sie mit Insulin behandelt sind – eine vermehrte Wasserretention aufweisen, ist eine zusätzliche Therapie mit niedrigdosierten Diuretika im Sinne einer Dreifachkombination häufig sinnvoll.

In einer Blutdruckzielwert-Studie für die Sekundärprävention an 29.000 Patient:innen mit Typ 2 Diabetes in Deutschland und Österreich [44] erreichten 67 % der Patient:innen nach Herzinfarkt den Blutdruckzielwert 130/80 mm Hg und 90 % der Patient:innen nach Schlaganfall den Zielwertbereich 120/70–140/90. In einer kanadischen Studie erreichten nur 36 % der von praktischen Ärzten betreuten Diabetes-Patient:innen einen Zielwert von < 130/80 mm Hg [45].

Mancia und Grassi [4] berichteten, dass in prospektiven randomisierten Studien bei hypertensiven Diabetes-Patient:innen nur in 5 von 13 Studien systolische Blutdruckzielwerte ≤ 140 und nur in 6 von 13 Studien diastolische Blutdruckzielwerte ≤ 80 mm Hg erreicht wurden.

Bei Patient:innen mit inadäquat kontrollierten Blutdruckwerten trotz einer Dreifachkombination antihypertensiver Wirkstoffe, die auch ein Diuretikum einschließt, soll die zusätzliche Gabe eines Mineralokortikoidantagonisten zur Blutdruckkontrolle erwogen werden. Unter dieser Kombinationstherapie sind allerdings regelmäßige Kontrollen der Nierenfunktion und des Kaliums unabdingbar. In der FIDELIO Studie [46] brachte die zusätzliche Gabe von Finerenon zu ACE-Hemmer/Angiotensin Rezeptor Antagonist bei Patient:innen mit Typ 2 Diabetes und chronischer Nierenerkrankung eine signifikante Reduktion renaler wie auch kardiovaskulärer Ereignisse.

## Blutdrucksenkung durch Antidiabetika

Bei Verwendung von mehreren Antidiabetika-Klassen kommt es zu einer signifikanten Blutdrucksenkung, wodurch die Blutdruckeinstellung der Patient:innen verbessert wird. Während unter Metformin, Insulin, Sulfonylharnstoffen und DPP-4-Inhibitoren keine Veränderung der Blutdruckwerte beobachtet wurde, senken SGLT-2-Inhibitoren, GLP‑1 Rezeptoragonisten und Pioglitazon den Blutdruck signifikant.

In einer Metaanalyse von Qayyum [47] an 13 Studien senkten die Glitazone im Vergleich zu Placebo den systolischen/diastolischen Blutdruck signifikant um 4,9/1,8 mm Hg.

In einer Metaanalyse von Wang et al. [48] wurde der blutdrucksenkende Effekt von GLP‑1 Rezeptoragonisten in 13 randomisert kontrollierten Studien (RCTs) analysiert. Exenatide senkte im Vergleich zu Placebo den systolischen/diastolischen Blutdruck um 5,2 und 5,4 mm Hg und Liraglutid senkte den systolischen Blutdruck um 5,6 mm Hg.

In einer Metaanalyse von Baker et al. [49] an 6 Studien, in denen unter einer Therapie mit SGLT2-Inhibitoren 24-Stunden Blutdruckmessungen durchgeführt wurden, fanden sich signifikante Blutdrucksenkungen systolisch um 3,8 mm Hg und diastolisch um 1,8 mm Hg. Die Blutdrucksenkung während des Tages betrug 4,3 mm Hg und während der Nacht 2,6 mm Hg. Die Blutdrucksenkungen waren unter den 4 getesteten SGLT-2-Inhibitoren (Canagliflozin, Dapagliflozin, Empagliflozin und Ertugliflozin) nicht signifikant unterschiedlich und korrelierten auch nicht mit dem Ausgangsblutdruck oder der Gewichtsabnahme. Der blutdrucksenkende Effekt der SGLT-2-Inhibitoren ist bezüglich der Nephroprotektion und Kardioprotektion prinzipiell als günstig zu beurteilen.

In der EMPA-REG Outcome Studie [50], in der CANVAS-Studie [51] und in DECLARE [52] lagen die Ausgangsblutdrucke bei 135/77 mm Hg, 136/78 mm Hg und 135/85 mm Hg; es wurden ca. 80–90 % der Patient:innen mit einem oder mehreren Antihypertensiva behandelt. Erwartungsgemäß sank der Blutdruck in diesen Studien unter den SGLT-2-Inhibitoren signifikant weiter ab. In der EMPA-REG Outcome Studie kam es unter Empagliflozin zu einer hochsignifikanten Senkung von kardiovaskulärem Tod, Gesamtmortalität, und Progression der Nierenerkrankung, der Blutdruckabfall von BP 135/77 mm Hg auf 131/75 mm Hg hatte keinerlei negativen Effekt. Diese Daten weisen darauf hin, dass Blutdruckwerte um 130 mm Hg auch bei Hochrisikopatient:innen mit Typ 2 Diabetes nicht gefährlich sein dürften. Bei älteren Patient:innen sollte bei einer Kombinationstherapie von Antihypertensiva und SGLT-2-Inhibitoren eine häufigere Blutdruckmessung erfolgen, um im Bedarfsfall die Zahl oder Dosis der Antihypertensiva zu reduzieren.

## Blutdruckziele bei Diabetes mellitus je nach Komorbiditäten

### Blutdruckzielwerte und antihypertensive Therapie bei alten Patient:innen mit Diabetes

Es existieren nur wenige Studien, die speziell ältere Patienten mit Diabetes mellitus eingeschlossen haben, obwohl 30–60 % aller Diabetes-Patient:innen in der Welt älter als 65 Jahre sind [53] Ältere Patienten stellen eine sehr heterogene Gruppe dar, nicht wenige Patienten zwischen 65–75 Jahren sind frei von Komplikationen, generell ist aber die Komorbidität bei diesen Patienten deutlich erhöht (vor allem Herzinsuffizienz und Nierenerkrankung). Die STEP Studie [54] schloss die 8511 chinesische Patient:innen im Alter von 60–80 Jahren ein, die zu einem systolischen Zielblutdruck von 110–130 versus zu einem systolischen Zielblutdruck von 130–150 mm Hg randomisiert wurden. Die erreichten mittleren Blutdruckwerte lagen bei 128 vs. 136 mm Hg. Der recht breit definierte primäre Endpunkt kardiovaskulärer Ereignisse wurde mit der stärkeren Blutdrucksenkung um 24 % signifikant gesenkt. Knapp 20 % der Patient:innen in dieser Studie hatten Diabetes; Subgruppenanalysen nach Diabetes-Status liegen nicht vor. Bei komplexen und gebrechlichen Patient:innen können etwas höhere Zielwerte (< 150/90 mm Hg) angestrebt werden, bei Patient:innen ohne Komplikationen empfehlen die Guidelines der ADA einen Zielwert von 140/90 [25], jene von Kanada allerdings < 130/80 mm Hg [53]. Die ESC Guidelines zur kardiovaskulären Prävention [29] empfehlen systolische Blutdruckwerte von 130–120 mm Hg für Patient:innen mit einem Alter bis zu 69 Jahren und von < 140 (und herunter bis zu 130 mm Hg, wenn dies vertragen wird) für Patient:innen ≥ 70 Jahre.

### Nierenerkrankung

Eine internationale Expertengruppe hat für KDIGO (Kidney Disease Improving Global Outcomes) evidenzbasierte Richtlinien für die antihypertensive Behandlung von Diabetes-Patient:innen mit chronischer Nierenerkrankung festgelegt, die 2021 in überarbeiteter Version veröffentlicht wurden [48]. Es wird hier für Patient:innen mit chronischer Niereninsuffizienz ein strenger Zielwert für den systolischen Blutdruck von < 120 mm Hg, sofern toleriert gefordert [55], wobei diese Empfehlung kontrovers diskutiert wird [56].

Verschiedene Studien haben gezeigt, dass unterschiedliche Blutdruckzielwerte unterschiedliche Effekte auf Endorganschäden ausüben [18, 57] Während möglichst niedrige Blutdruckwerte für die Schlaganfallprävention [18] und zur Verhinderung der Progression der diabetischen Nierenerkrankung [57] sehr günstig sein dürften, werden kardiovaskuläre Ereignisse insgesamt oder auch die Sterblichkeit dadurch nicht gesenkt. Unter besonders niedrigen Blutdruckwerten (sowohl systolisch als auch diastolisch) wurden sogar signifikant mehr kardiovaskuläre Ereignisse beobachtet, sodass die vorliegende Leitlinie von niedrigeren Zielwerten als 130/80 mm Hg im Allgemeinen abrät. In der ROADMAP-Studie [58] wurde etwa mittels einer aggressiven Blutdrucksenkung (Zielblutdruck < 130/80 mm Hg) das Auftreten einer Mikroalbuminurie zwar signifikant verzögert, gleichzeitig stiegen aber die fatalen kardiovaskulären Ereignisse signifikant an, wobei dies primär auf vermehrte kardiovaskuläre Todesfälle bei Patient:innen mit präexistenter KHK zurückzuführen war.

In einer Metanalyse von 24 RCTs an nahezu 70.000 Patient:innen (14 Studien mit Patient:innen mit Diabetes und 10 Studien mit Patient:innen ohne Diabetes) wurde unter einer Blutdrucksenkung von 10/5 mm Hg eine hochsignifikante Reduktion der dialysepflichtigen Niereninsuffizienz (ESKD) um 21 % nachgewiesen mit einer Number-Needed-to-Treat (NNT) von nur 8 Patient:innen bei einer Therapiedauer von 5 Jahren [59] Bemerkenswert ist allerdings der hochsignifikante Unterschied (*p* < 0,0001) zwischen Patient:innen mit Diabetes (HR 0,79 (0,66–0,95)) und ohne Diabetes (HR 1,01 (0,81–1,26)). Diese Metaanalyse würde also nahelegen, dass eine Blutdrucksenkung bei Patient:innen ohne Diabetes keine Senkung des Risikos einer dialysepflichtigen Niereninsuffizienz bewirkt, während bei Patient:innen mit Diabetes mellitus eine relativ kleine Blutdrucksenkung von 10/5 mm Hg eine dramatische Verbesserung bewirken könnte.

Allerdings waren sowohl in ACCORD-BP als auch in SPRINT-Studie [35] Patient:innen im intensivierten Arm einem höheren Risiko für das Entstehen einer CNI (eGFR > 30 % auf < 60 mL/min per 1,73 m^2^) ausgesetzt, als im Standard-Arm. Eine Überwachung der Nierenfunktion unter intensivierter Blutdruckkontrolle erscheint daher sehr wichtig.

### Schlaganfall

Xie et al. [60] analysierten den Effekt einer Blutdrucksenkung auf das Schlaganfall-Risiko in einer Network Analyse aus 21 RCTs, die insgesamt 65.000 Patient:innen eingeschlossen hatten. Untersucht wurde die Risikosenkung einer Blutdrucksenkung von 10/5 mm Hg bei systolischen Ausgangsblutdrucken > 140, < 140, > 130 und < 130 mm Hg. Für Patient:innen mit den niedrigsten Ausgangsblutdrucken (< 130 mm Hg) fand sich noch immer eine signifikante Schlaganfall-Reduktion bei Diabetes-Patient:innen (HR 0,61 [95 %CI: 0,47–0,79]).

In der ACCORD-BP Studie [18] wurde unter der intensivierten Blutdrucksenkung mit einem Zielwert von 119,5 mm Hg im Vergleich zum Kontrollarm (133,5 mm Hg) ebenfalls eine hochsignifikante Risikoreduktion des Schlaganfalls (HR 0,59 [95 % CI: 0,39–0,89]) beobachtet.

### PAVK

Diabetes-Patient:innen mit PAVK haben eine weitaus schlechtere Prognose als Diabetes-Patient:innen nach Herzinfarkt oder Schlaganfall, wie in der PROactive-Studie nachgewiesen werden konnte [61]; eine rezente österreichische Studie zeigt ähnliche Ergebnisse [62]. Früher wurden für PAVK-Patient:innen generell Blutdruckzielwerte < 130/80 mm Hg gefordert, obwohl dafür keine Evidenz aus Studien vorlag. In der posthoc Analyse der INVEST-Studie [63] wurde das besonders hohe Risiko der PAVK-Patient:innen (Diabetesanteil 41 %) bestätigt. Bei insgesamt 2699 Patient:innen mit PAVK (1106 Patient:innen mit Typ 2 Diabetes) zeigte sich nach einer Studiendauer von 2,6 Jahren ein enger Zusammenhang zwischen den Blutdruckwerten und dem primären Endpunkt (Gesamtmortaliät, nichtfataler Herzinfarkt und nichtfataler Schlaganfall). Der beste Outcome (niedrigste HR für den primären Outcome) fand sich bei systolischen Blutdruckwerten zwischen 135 bis 145 mm Hg und diastolischen Werten zwischen 60–90 mm Hg. Bei niedrigeren systolischen Blutdruckwerten stieg die HR bis auf 1,7 (systolischer Blutdruck um 110 mm Hg) an, bei Blutdruckwerten um 180 mm Hg lag die HR sogar bei 2,5. Insgesamt ist die Datenlage bei PAVK und Diabetes mellitus besonders schwierig, da es wenig Primärstudien gibt.

### KHK

Verschiedene Studien legen nahe, dass insbesondere bei Diabetes-Patient:innen mit gleichzeitig bestehender KHK das Blutdruckziel nicht < 130 mm Hg systolisch liegen sollte. So zeigt etwa eine Auswertung von Daten der SAVOR TIMI 53 Studie [39] das niedrigste kardiovaskuläre Risiko bei Blutdruckwerten von 130–140 mm Hg systolisch und von 80–90 mm Hg diastolisch. Da allerdings sehr viele Diabetes-Patient:innen diese Blutdruckzielwerte trotz Einsatz mehrerer Antihypertensiva bei weitem nicht erreichen, ist im klinischen Alltag die Angst vor zu niedrigen Blutdruckwerten wahrscheinlich wesentlich unbedeutender als das Bemühen systolische Blutdruckwerte unter 140 mm Hg zu erreichen.

## Metaanalysen und Expertenmeinungen

Es sind mehrere Metaanalysen zur antihypertensiven Therapie bei Diabetes publiziert worden. Die zum Teil widersprüchlichen Ergebnisse machen es verständlich, warum verschiedene Fachgesellschaften unterschiedliche Blutdruckzielwerte empfehlen. Aggarwal et al. [64] analysierten den Effekt von Blutdruckzielwerten < 120/80 mm Hg versus < 140/mm Hg an 14.000 Patient:innen mit und ohne Diabetes aus den kontrollierten Studien von ACCORD-BP und SPRINT. Patient:innen mit den niedrigen Blutdruckzielwerten hatten signifikant weniger CV-Ereignisse (HR 0,83; 95 % CI, 0,74–0,92; *P* < 0,001), eine Interaktion mit Diabetes war nicht nachweisbar, wenngleich die positiven Effekte einer intensiven Blutdrucksenkung bei den Patient:innen ohne Diabetes viel stärker ausgeprägt waren. Thomopolous et al. [59] analysierten das Ausmaß einer Blutdrucksenkung von 10/5 mm Hg in 27 RCTs bei Patienten mit Diabetes und in 28 RCTS bei Patient:innen ohne Diabetes in Abhängigkeit vom Ausgangsblutdruck (> 140, 130–140 und < 130 systolisch). Während die Blutdrucksenkung auch bei niedrigen Ausgangsblutdruckwerten bei Patient:innen ohne Diabetes für alle Endpunkte positive Effekte zeigte, fand sich dies bei Diabetes-Patient:innen nur für den Endpunkt Schlaganfall, ein signifikanter Anstieg von Gesamtmortalität und kardiovaskulärem Tod wurde allerdings nicht beobachtet. Aus einer weiteren Metaanalyse hat dieselbe Arbeitsgruppe den Schluss gezogen, dass Blutdruckwerte um 130 mm Hg mit dem geringsten vaskulären Risiko assoziiert sein dürften [65], eine ganz ähnliche Beurteilung kommt von der Arbeitsgruppe von Sarafidis [66]. Eine umfangreiche Analyse von Mancia und Grassi [4] schließt mit der Feststellung, dass die Blutdruckzielwerte bei Diabetes-Patient:innen wahrscheinlich niedriger als < 140/90 sein sollten und wahrscheinlich 130/80 mm Hg betragen sollten. Die Evidenz für Blutdruckzielwerte < 130/80 mm Hg ist nach Meinung dieser Experten nicht ausreichend, Blutdruckzielwerte < 120/80 mm Hg sollten aber vermieden werden. In einer rezenten Meta-Analyse, die Daten von über 340.000 Studienteilnehmer:innn verwendet, war allerdings, ohne dass hier das Vorliegen eines Diabetes berücksichtig worden wäre, eine 5 mm Hg Senkung des systolischen Blutdrucks mit einer Risikoreduktion schwerer kardiovaskulärer Ereignisse um 11 % (bei vorbestehender kardiovaskulärer Erkrankung) bzw. 9 % (ohne vorbestehende kardiovaskuläre Erkrankung) assoziiert – unabhängig vom systolischen Ausgangsblutdruck [67].

## Zusammenfassung

Internationale Fachgesellschaften empfehlen für Diabetes-Patient:innen unterschiedliche Blutdruckzielwerte < 140/90 mm Hg (ADA) oder < 130/80 mm Hg (AHA, ESC).

Ähnlich wie bei der Blutzuckereinstellung erscheint es in Anbetracht der Evidenzlage sinnvoll, eine Individualisierung der Blutdruckzielwerte in Abhängigkeit von Alter und Komorbiditäten (Schlaganfall, Herzinfarkt, Herzinsuffizienz, PAVK, Nierenerkrankung) anzustreben. Besonders wichtig ist, dass zumindest ein Blutdruck < 140/90 mm Hg erzielt wird; lediglich bei gebrechlichen, multimorbiden alten Menschen erscheint ein Ziel < 150/90 mm Hg vertretbar.

Patient:innen mit bestimmter Komorbidität – nach Schlaganfall oder bei Vorliegen einer Nierenerkrankung – profitieren eindeutig von niedrigeren Zielwerten, sodass es auf Basis der gegenwärtigen Evidenz am sinnvollsten erscheint, eine Individualisierung der Blutdruckeinstellung bei Patient:innen mit Diabetes mellitus anzustreben. Ein Zielblutdruck um 130/80 mm Hg dürfte nach gegenwärtiger Evidenz jener sein, der mit der relativ besten Prävention von vaskulären Komplikationen und mit der größten Sicherheit einhergeht. Eine Überwachung der Nierenfunktion unter intensivierter Blutdruckkontrolle ist auf jeden Fall sehr wichtig.

Niedrigere Zielwerte (120/80 mm Hg) sind für die Primär-und Sekundärprävention von Schlaganfall und Nierenerkrankung prinzipiell günstig, wegen des erhöhten Auftretens von schwerwiegenden Nebenwirkungen ist allerdings Vorsicht angezeigt. Blutdruckzielwerte unter 120 mm Hg systolisch und unter 70 mm Hg diastolisch können aufgrund der potenziellen Steigerung der Mortalität keineswegs empfohlen werden.

## Interessenkonflikt

C.H. Saely hat in Bezug auf diesen Artikel von folgenden Unternehmen, die teils auch fördernde Mitglieder der ÖDG sind, Forschungsunterstützungen und/oder Honorare erhalten: MSD, AstraZeneca, Amgen, Sanofi-Aventis, Boehringer-Ingelheim, Novartis, Pfizer, Genericon, Takeda, Bayer. G.-H. Schernthaner gibt an, dass kein Interessenkonflikt besteht. J. Brix: Honorare für Advisory Boards und Vorträge von Astra Zeneca, Boehringer-Ingelheim, Eli Lilly, Novo Nordisk, Sanofi-Aventis. R. Klauser-Braun hat in Bezug auf diesen Artikel von folgenden Unternehmen, die teils auch fördernde Mitglieder der ÖDG sind, Forschungsunterstützungen und/oder Honorare erhalten: AstraZeneca, Böhringer Ingelheim, Kwizda, Menarini, Merck Sharp Dome, Novartis, Pfizer, Ratiopharm, Sanofi-Aventis, Servier, Takeda. E. Zitt hat in Bezug auf diesen Artikel von folgenden Unternehmen, die teils auch fördernde Mitglieder der ÖDG sind, Forschungsunterstützungen und/oder Honorare erhalten: AstraZeneca, Boehringer-Ingelheim. H. Drexel hat in Bezug auf diesen Artikel von folgenden Unternehmen, die teils auch fördernde Mitglieder der ÖDG sind, Forschungsunterstützungen und/oder Honorare erhalten: Amgen, AstraZeneca, Bayer Austria, Boehringer-Ingelheim, Meda Pharma, Merck Sharp & Dohme, Novo Nordisk, sanofi-aventis, Servier. G. Schernthaner gibt an, dass kein Interessenkonflikt besteht.
